# Breast cancer regulated by Fringe

**DOI:** 10.18632/oncoscience.203

**Published:** 2015-08-20

**Authors:** Keli Xu

**Affiliations:** Cancer Institute and Department of Neurobiology and Anatomical Sciences, University of Mississippi Medical Center, Jackson, Mississippi, USA

**Keywords:** breast cancer, Fringe, Notch

Breast cancers are heterogeneous at clinical and molecular levels. In recent years gene expression profiling has been used to classify breast cancers into at least five molecular subtypes, including basal-like, claudin-low, luminal A/B, HER2-enriched and normal-like. Among them, basal-like and claudin-low subtypes account for the majority of triple negative breast cancer (TNBC), a group of diseases that are defined on the basis of being negative for estrogen receptor, progesterone receptor and human epidermal growth factor receptor 2 [1]. TNBC preferentially affects younger patients, is more prevalent in women with African ancestry, and is often more aggressive than other types of breast cancer. Basal-like breast cancer (BLBC) expresses markers of myoepithelium/basal cells and shares features with bipotent progenitor cells, and is thought to have originated from mammary bipotent/luminal progenitor cells. Claudin-low breast cancer (CLBC) shares more features with mammary stem cells and cells that have undergone epithelial-to-mesenchymal transition.

Fringe proteins are β3N-acteylglucosaminyl-transferases that are known to modify EGF repeats in the extracellular domains of Notch receptor thereby modulating ligand-mediated Notch activation [2]. There are three Fringe genes in mammals, namely, *Lunatic Fringe (Lfng)*, *Manic Fringe (Mfng)*, and *Radical Fringe (Rfng)*. In the mouse mammary gland, *Lfng* expression is restricted to the stem/progenitor cell-enriched cap cell layer of the terminal end bud [3]. Deletion of *Lfng* in mouse mammary epithelium (*Lfng*flox/flox*;MMTV-Cre*) resulted in ectopic proliferation, expansion of the basal compartment, and ultimately, development of mammary tumors, in which two-thirds resemble BLBC and one-third are similar to CLBC. In agreement with the mouse experiment, the vast majority of BLBC and a subset of CLBC in humans exhibit significantly decreased *LFNG* expression [3].

While *Lfng* deficiency is a hallmark of BLBC, we recently reported that *Mfng* is highly expressed in CLBC and functions as an oncogene in this context [4]. We showed that Mfng regulates Notch activation in human and mouse CLBC cell lines, as well as in mouse mammary gland. Knockdown of *Mfng* in CLBC cell lines reduced cell migration and tumorsphere formation associated with a decrease in the stem-like cell population, as well as diminished *in vivo* tumorigenicity. Deletion of *Mfng* in *Lfng*flox/flox*;MMTV-Cre* mice caused a tumor subtype shift away from CLBC. We identified the phosphoinositide kinase Pik3cg as a direct transcriptional target of Mfng-facilitated RBPJk-dependent Notch signaling in CLBC cells. *PIK3CG* is aberrantly expressed in many invasive human breast tumors and its expression level correlates with metastatic potential of breast cancer cell lines [5]. We showed that pharmacologic inhibition of PI3Kγ in CLBC cell lines blocked migration and tumorsphere formation, suggesting that Mfng-induced Pik3cg contributes to breast cancer aggressiveness by promoting cell migration and invasion, as well as maintaining cancer cell stemness. Identification of Pik3cg as a Notch target prompts a PI3Kγ-targeting strategy for the treatment of CLBC and perhaps other poor prognosis breast cancers. Interestingly, Met proto-oncogene was amplified in both basal-like and claudin-low subtypes of the *Lfng*flox/flox*;MMTV-Cre* mammary tumors [3], and Met synergized with p53 loss to induce mouse mammary tumors resembling CLBC [6]. Therefore combination targeting of both Met and PI3Kγ may prove beneficial for the treatment of this disease. Indeed, we observed synergistic effects of Met and PI3Kγ inhibitors on growth of multiple human CLBC cell lines (Chung and Xu, unpublished data).

MFNG expression in human breast cancer is highly correlated with the expression of NOTCH4, but not other Notch receptors. In addition, *Mfng* silencing in CLBC cell lines consistently decreased Notch4 activation, while *Mfng* deletion in the mouse mammary gland resulted in decreased Notch4 activation [4]. Thus, Mfng appears to control primarily Notch4-mediated signaling in the mammary epithelium. Given that Notch4 is enriched in mammary stem cells, a putative cell-of-origin of CLBC, the Mfng-Notch4-Pik3cg signaling cascade may be a driving force for CLBC pathogenesis. Next question is what regulates Mfng in this context. To this end, we have found that overexpression of Myc upregulates Mfng in mouse mammary epithelium as well as in human breast cancer cell lines (Zhang and Xu, unpublished data). Intriguingly, Myc was found amplified in almost half of human CLBC cases [7]. A link between Myc and Mfng during CLBC development warrants further investigation.

Different subtypes of breast cancer are thought to have different cellular origins. Notch pathway may utilize different Fringes to precisely control signaling in distinct cell types of mammary epithelial hierarchy. Altered Fringe expression/activities may cause dysregulation of Notch signaling in different cells-of-origin, and ultimately, leading to breast cancer of different subtypes. Lfng inhibits Notch activation in luminal progenitor cells to prevent BLBC, whereas Mfng may enhance Notch4 signaling in mammary stem cells to promote CLBC initiation and progression. Of note, loss of both *Lfng* and *Mfng* in the mammary epithelium dramatically decreased activation of multiple Notch receptors and induced adenosquamous carcinoma [4], suggesting a redundant role of two genes in certain mammary cell types.
